# Architecture design of TiO2 with Co-doped CdS quantum dots photoelectrode for water splitting

**DOI:** 10.55730/1300-0527.3604

**Published:** 2023-09-30

**Authors:** Fatih TEZCAN, Abrar AHMAD, Gülfeza KARDAŞ

**Affiliations:** 1 Department of Chemistry and Chemical Process Technology, Vocational School of Technical Sciences at Mersin Tarsus Organized Industrial Zone, Tarsus University, Mersin, Turkiye; 2 Department of Chemistry, Faculty of Arts and Science, Çukurova University, Adana Turkiye; 3 Department of Chemistry, Quaid-i-Azam University, Islamabad, Pakistan

**Keywords:** TiO_2_, hydrogen production, photoanode, CdS QDs

## Abstract

Photoelectrochemical hydrogen production is a critical key to solving the carbon-zero goal of countries due to renewable sources of solar light and combustion products of hydrogen-only water. Here, an architecture design for an n-type nano rosettes-rod TiO_2_ (RT) surface using CdS and Co-doped CdS quantum dots (QDs) is carried out utilizing the SILAR (simple ionic layer adsorption and reaction) method. Furthermore, the photocatalytic behaviour of Co-doped CdS QDs SILAR cycles deposition is investigated in various cycles, including 5, 8, 10, and 12. The FESEM, Raman XRD, Uv-Vis spectrometer, and vibration modes are used to evaluate the photoelectrode surface structure, crystal structure, and solar light absorption, respectively. FESEM images and XRD pattern revealed successive CdS QDS and Co-doped CdS QDs deposition on the RT boundary and rising SILAR cycles of Co-doped CdS QDs lead to further coverage of RT surface. UV-vis spectrometer indicated shifting solar light absorption to the visible region by applying more SILAR cycles of Co-doped CdS QDs deposition. The electrochemical parameters obtained from EIS showed total polarization resistance (R_p_) of the RT electrode dramatically decreased with 10 SILAR cycle Co-doped CdS QDs deposition (5093 Ω cm^2^ and 617 Ω cm^2^). Linear sweep voltammetry (LSV) and chronoamperometric photocatalytic performance measurements indicated Co-doped CdS QDs on RT extremely enhanced photoresponse under solar irradiation and 10 SILAR cycle Co-doped CdS QDs improved photocurrent density about fourfold according to blank RT electrode.

## 1. Introduction

The intensive use of fossil-based resources in energy production for centuries has brought us to irreversible climate change [1–3]. At this level, countries have been announcing short-term and long-term carbon-zero action plans [4,5]. Action plans are created within environmentally friendly energy production and sustainability using renewable energy sources. According to this perspective, solar energy is the most critical potential in renewable energy sources. Solar energy can be transformed into electrical or direct photon energy. Photoelectrochemical hydrogen gas production uses photon energy from direct sunlight and applies bias potential by water splitting [6–9].

In the literature, the various semiconductors have been studied in the production of photoelectrochemical hydrogen gas such as ZnO [10,11], WO_3_[12,13], Cu_2_O [14,15], CuO [16–18], and TiO_2_ [19–22]. TiO_2_, an n-type semiconductor, is widely preferred because it can be easily synthesized, modified in various morphologies, is chemically stable and is eco-friendly [23]. However, the absorption of TiO_2_ in the UV region causes its utilization from sunlight to be relatively low [24]. Surface modifications on TiO_2_ have been performed to improve solar light efficiency by shifting to the solar spectrum’s visible region. Heterostructure application is mainly conducted on wide (E_g_) semiconductors by low Eg bandgap semiconductors [25–27]. A n-type semiconductor CdS, which can be synthesized as quantum dots (QDs), is widely preferred for constructing heterostructures in electrodes with large E_g_ values [28–30]. Furthermore, the absorption of CdS QDs performs with multiple excitations to solar-driven electron production in photocatalytic applications [31]. Therefore, electron transfer to wide E_g_ electrode can be enhanced with CdS QDs heterostructure. In addition, Mn, Cu, and Co metals doped to CdS QDs can be promising to improve photocatalytic performance [32–36]. Especially, many papers published about Mn-doped CdS QDs due to the increase of electron/hole lifetime for electron transition among broad band gap semiconductor energy levels for solar cells and photoelectrochemical cells [37,38]. Although CdS QDs modified TiO_2_ application is more studied, Co-doped CdS QDs application is limited to solar and photoelectrochemical cells, especially on the TiO_2_ substrate. For instance, Zou and co-workers [39] studied CdS/Co-doped CdSe electrodes on solar cells, suggesting Co-doping to CdSe, dropping the recombination of electron-hole, thus enhancing cell efficiency. Meng et al. [40] synthesized Co-doped carbon quantum dots on CdS nanorods (NRs) with various loading by hydrothermal deposition method for photocatalytic hydrogen production. It demonstrated exceptional photocatalytic activity due to the superior synergistic consequence between the Co nanoparticles and the carbon quantum dots. Co-doped CdS nanoparticles may generate a redshift, according to Thambidurai and colleagues [41], the chemical precipitation method produced CdS quantum dots. In the current study, we studied the design of a three-dimensional RT surface architecture using CdS QDs and Co-doped CdS QDs with the procedure of the simple ionic SILAR, which is inexpensive and fast to implement because it has no connection to a device and is extremely basic. Additionally, the photocatalytic behavior of Co-doped CdS QDs SILAR cycles deposition is investigated in various cycles, covering 5, 8, 10, and 12.

## 2. Materials and methods

### 2.1. Materials

Ti(OC_4_H_9_)_4_ (97%), TiCl_4_ (≥99.0%), Cd(NO_3_)_2_.4H_2_O, (98%), (CH_3_COO)_2_Co.4H_2_O (99.0%), Na_2_S.9H_2_O (≥98.0%), HCl (37%), Na_2_SO_3_ (95%), C_3_H_6_O (99.5%) C_2_H_5_OH (99.9%) were utilized without any further purification. The photocatalyst was synthesized on a Fluorine-doped tin oxide (FTO, ~8 Ω/Sq). The FTO working electrode was cleaned in the ultrasonic bath using a detergent solution, C_3_H_6_O, C_2_H_5_OH, and distilled water for a total of 10 min respectively.

### 2.2. Synthesis of the nano rosettes-rod TiO_2_

Synthesis of 3D nano rosettes-rod TiO_2_ (RT) consists of two synthesis procedures according to a previous study by hydrothermal deposition method [42]. The first step is 1D TiO_2_ hydrothermally synthesis by an earlier applied procedure. Concentrated HCl and distilled water were gradually mixed with similar volume and solution with constant stirring for 5 min. Ti(OC_4_H_9_)_4,_ was slowly put in solution, which was poured into a Teflon-lined stainless steel autoclave that previously replaced FTO substrates (surface area of 1 cm^2^). The synthesis of TiO_2_ was conducted for 12 h at 150 °C in an oven by hydrothermal deposition. After deposition, electrodes were cleaned repeatedly with distilled water and C_2_H_5_OH before being dried in an electric oven at 60 °C. The electrodes were then heated in the furnace for an h at 500 °C.

In the second step, RT was obtained from the synthesis TiO_2_ electrode. An autoclave at 100 °C for one h with a concentrated TiCl_4_ solution. The same quantity of strong HCl and distilled water were added and stirred for 5 min. Step-by-step pours of concentrated TiCl_4_ were performed, and the mixture was stirred once again for a duration of five min. Then the solution was replaced in an autoclave, which previously added TiO_2_ electrode. The hydrothermal deposition was carried out in an autoclave for three h at 150 °C. The electrodes were washed with distilled water and ethyl alcohol before being heated to 60 °C. Finally, RT was heated for one h at a temperature of 500 °C.

### 2.3. Synthesis of Co-doped CdS on RT

CdS QDs and Co-doped CdS QDs were synthesized by the SILAR method. Initially, the samples were immersed in 0.1 M Cd (NO_3_)_2_.4H_2_O for 5 min, allowing Cd^2+^ to diffuse to the RT surface and adsorb there. The RT sample was then removed from the solution and rinsed with distilled water to remove extra Cd^2+^ cation. CdS QDs were produced by allowing the S^2^^−^ anion to adsorb on the Cd^2+^ terminated RT surface after the sample was exposed to 0.1 M Na_2_S.9H_2_O for 5 min, it is represented as RTC. The RTC sample was washed with distilled water and dried with an N_2_ purge. The technique described above is known as a SILAR cycle. Co dopant in CdS QDs synthesis was performed in (CH_3_COO)_2_Co.4H_2_O-Cd (NO_3_)_2_.4H_2_O precursor solution. According to the experimental setup, both Co^2+^ and Cd^2+^ cations chemically adsorb to S^2^^−^ on the RT surface. The SILAR cycles 5, 8, 10, and 12 were referred to as RTCCo_xCy (where x varies regarding 5, 8, 10, and 12 cycles).

### 2.4. Characterization of the Photoelectrode

The crystal structure and hkl parameters of the prepared electrodes were characterized using an X-ray diffraction (XRD) pattern (Malvern PANalytical Empyrean). The surface structure was investigated using a scanning electron microscope **(**FEI Quanta 650 Field Emission SEM**)**. The vibration modes of the semiconductor were studied at 532 nm excitation by Raman spectrophotometer (Renishaw InVia Qontor). A Uv-vis spectrometer (Agilent, Cary 7000) was utilized to obtain the optical absorption spectra of obtained photoelectrodes to characterize samples’ absorption behaviour and band gap (Eg).

### 2.5. Photoelectrochemical test

The photocatalytic performances were carried out using the widely used three-electrode systems. The reference electrode, working electrode, and counter electrode are saturated Ag/AgCl (3 M KCl), the synthesis electrodes, and a Pt sheet (2 cm^2^), respectively. There were 0.1 M Na_2_SO_3_ and 0.1 M Na_2_S in the solution. The CHI 660D electrochemical analyser was used to conduct linear sweep voltammetry (LSV), electrochemical impedance spectroscopy (EIS), and Mott-Schottky experiments. The LSV measurement varied from −1.2 to 0.6 V (vs. Ag/AgCl) at a scan rate of 5 mV s^−^^1^. As the irradiation source of 100 mW cm^−^^2^, a solar simulator (Sunlite TM sun Simulators, AM 1.5 filter) was utilized. EIS measurements were performed between 10^−^^1^ and 10^5^ Hz at 0.3 V_RHE_ over-potential, 5 mV in amplitude, and under 100 mW cm^−^^2^. EIS results were fitted with ZView software to derive electrochemical parameters.

Chronoamperometric measurement (j-t) was performed using a chopped technique interval of 20 seconds light at 0.3 V (vs RHE) over-potential for 5 min. Mott-Schottky experiments were taken in the −0.4–0.2 V (vs. RHE) range, with a 5 mV amplitude at 500 Hz frequency and no light circumstances. The reference hydrogen electrode (RHE) was normalized at the photocatalytic measured potential vs. Ag/AgCl reference electrode at the photocatalytic measured potential. The following equation was used to calculate the overall potential of the water-splitting reaction vs RHE [40];


(1) $$E_{RHE}=E_{Ag/AgCl}+E_{Ag/AgCl}^{\circ }+0.059pH$$

Herein, *E_RHE_* is converted potential vs. RHE, *E_Ag_*_/_*_AgCl_* is the obtained potential vs. Ag/AgCl reference electrode, and $E_{Ag/AgCl}^{\circ }$ is 0.197 V for 3.0 M KCl at 25 °C.

An applied bias photon-to-current efficiency (*η*) of the samples, that is, the photoconversion efficiency, was calculated according to the following equation:


(2) $$\eta (\%)=J_{ph}\frac{1.23-V}{p_{total}}\times 100$$

Where, *J_ph_* is the obtained photocurrent density, *V* is the over-potential vs RHE, and *p_total_* is used solar light (100 mW cm^−^^2^).

## 3. Results

### 3.1. Photoelectrode characterization

**Figure 1:**
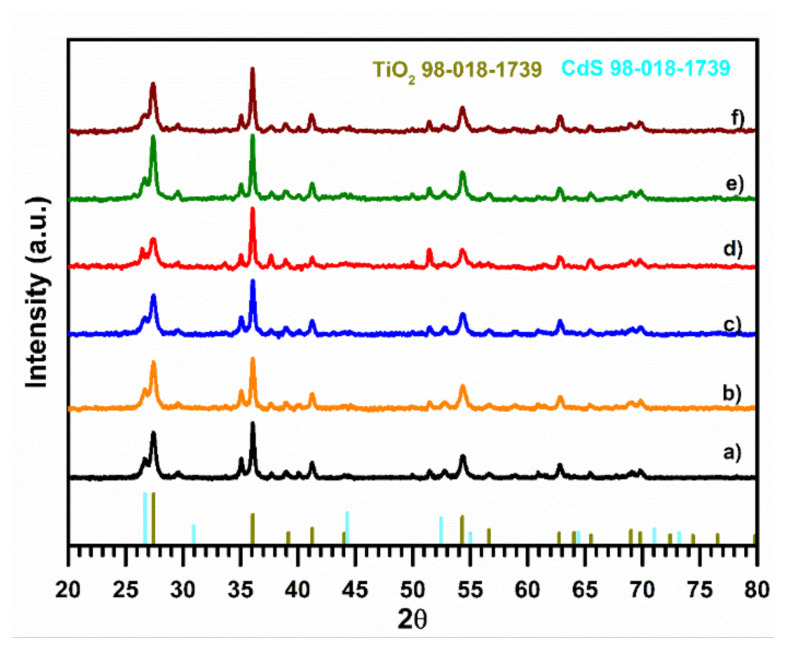
XRD pattern of (a) RT, (b) RTC, (c) RTCCo_5Cy (d) RTCCo_8Cy, (e) RTCCo_10Cy, and (f) RTCCo_12Cy.

The crystal structure and phase planes of the samples were measured with 2θ ° values ranging from 20 ° to 80 ° by XRD pattern. The XRD pattern of samples is depicted in Figure 1. The pattern of pristine RT exhibits 2θ ° values and hkl are 27.20° (110), 36.01 ° (101), 38.85 ° (200), 41.14 ° (111), 44.26 ° (210), 54.28 ° (211), 56.61 ° (220), 62.83 ° (002), 65.47 ° (221), and 69.01° (301). The prepared sample is the tetragonal rutile phase of TiO_2_ (pdf card no: 98-016-8140) [43]. Furthermore, the main peaks 27.20 °, 36.01 °, and 54.28 ° indicate successively obtained RT [44,45]. According to previous studies, CdS QDs synthesis with the SILAR method commonly obtained the cubic phase of CdS [46,47]. The main distinctive 20 ° peaks of CdS are 26.72 ° and 44.33 °, associated with the (111) and (202) hkl parameters. But these peaks overlap with 27.40 ° and 44.04 ° the rutile phase of TiO_2_, resulting in distinct 2θ ° values incredibly similar to the characteristic peaks of the sample and causing identifying difficulty of 2θ ° peaks [48,49]. Additionally, a considerable peak difference is unavailable in the XRD pattern with the addition of Co^2+^ and various SILAR cycles. It can be concluded that the nanoparticles adsorb on TiO_2_, leading to few amount depositions on TiO_2_ and causing under the measurement detection limit/overlap peak observation. Therefore, FESEM, Raman and Uv-vis analysis were performed to define the Co_-_doped CdS QDs on the RT surface.

**Figure 2:**
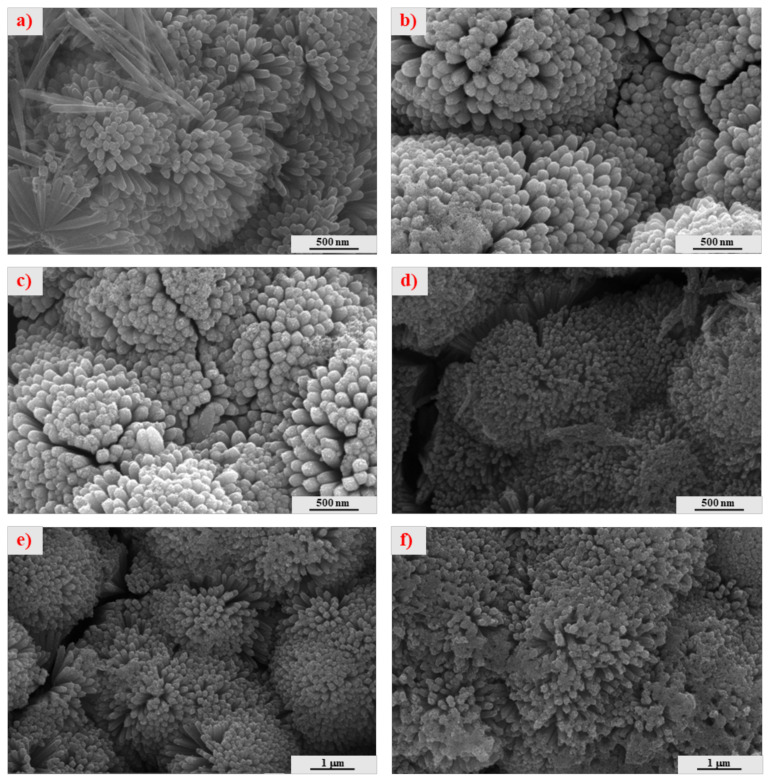
FESEM images (a) RT, (b) RTC, (c) RTCCo_5Cy, (d) RTCCo_8Cy, (e) RTCCo_10Cy, and (f) RTCCo_12Cy.

The surface morphology of RT_,_ RTC, and various RTCCo photoelectrodes are given in Figure 2. According to FESEM images, the nano rosettes clearly observe with the applied two synthesis setup procedure. RT consist of tiny nanorods play acting as a structural component. Therefore, at the second step of the synthesis, TiO_2_ grow up on the tip of the nanorod, enabling active surface boundary to adsorb Co-doped CdS QDs and enhancing solar light efficiency. In Figure 2 (b), CdS deposition causes QDs accumulation over RT, and its surface converts rougher surface structure. Furthermore, the nanoparticles are perfectly deposited on the RT surface and increasing SILAR cycle enhanced Co-doped CdS QDs deposition. Also, Co-doped CdS nanoparticles further attached the tip of RT at the 12 cycles, causing clog RT active surface and decreasing the RT surface area to performing the photoelectrochemical process (in Figure 2(b)).

**Figure 3:**
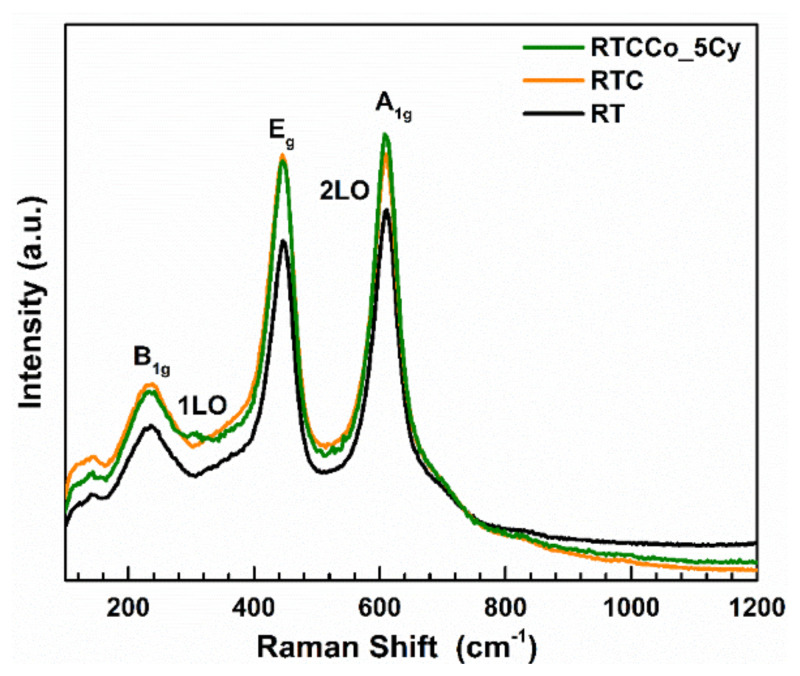
Raman spectrum of RT, RTC, RTCCo_5Cy.

Raman measurements to define vibration modes of CdS QDs and Co dopant CdS QDs on RT and further probe the content of the photoelectrode were taken between 100 and 1200 cm^−^^1^. Raman spectrums of the photoelectrodes are given in Figure 3. The pristine RT gives 237, 446, and 612 cm^−^^1^, associated with the main three B_1g_, E_g,_ and A_1g_ vibration modes of RT [50]. Both E_g_ and A_1g_ are related to the active mode, however, B_1g_ vibration mode corresponds to multiple phonon vibrations. In addition, in terms of RTCCo_5Cy photoanode, deposition of CdS on RT can be clearly confirmed with two peaks at 300 cm^−^^1^ and 606 cm^−^^1^, connected to the first order longitudinal optical phonon (1LO) and second order longitudinal optical phonon (2LO) on active Raman mode [51,52]. As seen in the Raman spectrum, 2LO of the CdS peak overlapped with A_1g_ of TiO_2_, but the 1LO peak obviously appeared at 300 cm^−^^1^, suggesting deposition of CdS.

**Figure 4:**
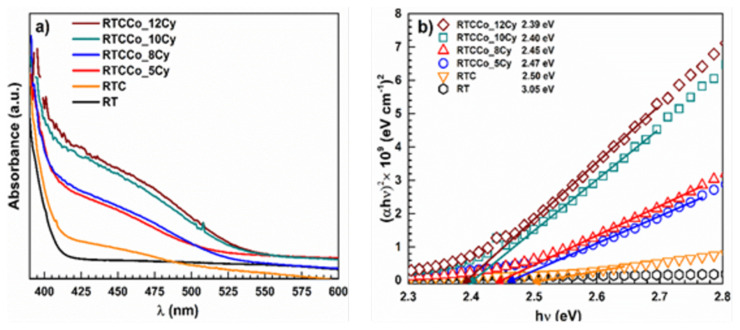
Uv-Vis spectrum (a) and Tauc plot (b) of the photoelectrodes.

A UV-visible spectrometer was used to examine the solar light absorption behaviour and band gap (E_g_) energy of electrodes. The solar absorption spectrum ranges from 380–800 nm of the pristine RT and various RTCCo electrodes are given in Figure 4. Commonly, the TiO_2_ electrode absorbs light in the ultraviolet region [53]. RT absorption inset value is about ~ 410 nm, and the E_g_ value is 3.05, consistent with previous TiO_2_ studies. As CdS QDs deposition on the RT surface, the absorption inset is shifted from ultraviolet to the visible region at about 520 nm. Furthermore, Co^2+^ doped CdS QDs also is shifted further visible region, suggesting Co^2+^ replaced among both valence and conduction band energy levels of CdS QDs and supplied further electron transfer [54,55]. Also, adding Co^2+^ in the CdS QDs, can be concluded that the Co^2+^ loading quantity of QDs enabled to increase in the deposition of QDs on the RT boundary. According to various RTCCo electrode spectrum, absorbance is shifting visible region with increasing SILAR cycles RTCCo electrodes and RTCCo_12Cy demonstrates absorption inset behaviour in the more visible region. It suggests that increasing the SILAR cycle enables further Co^2+^ loading quantity of CdS QDs and improves electron transportation along with the CdS energy level. In addition, E_g_ values of samples are calculated by Tauc’s following equation;


(3) $${\left(\alpha h\upsilon \right)}^{1/r}=A(h\upsilon -E_{g})$$

Herein, *α* is the absorption coefficient, *h* is a Planck constant, *υ* is frequency, *r* vis the direct band transition ( *r = 1/2*), and A is the experimental constant. The Uv-vis absorption curve converts to *(αhν)^2^* versus *hν*. E_g_ value is obtained by extrapolating the curve to the x-axis of the curve drawing, and Tauc’s plots are given in Figure 4 (b). According to E_g_ values, Co-doped CdS QDS enable lower E_g_ value compared with bare CdS QDs electrode, and the various Co-doped CdS QDs E_g_ is decreased with increasing SILAR deposition cycle numbers. It indicates that in large amounts deposition caused to boost of the recombination rate of photogenerated electron-hole pairs.

### 3.2. Photoelectrochemical performance

**Figure 5:**
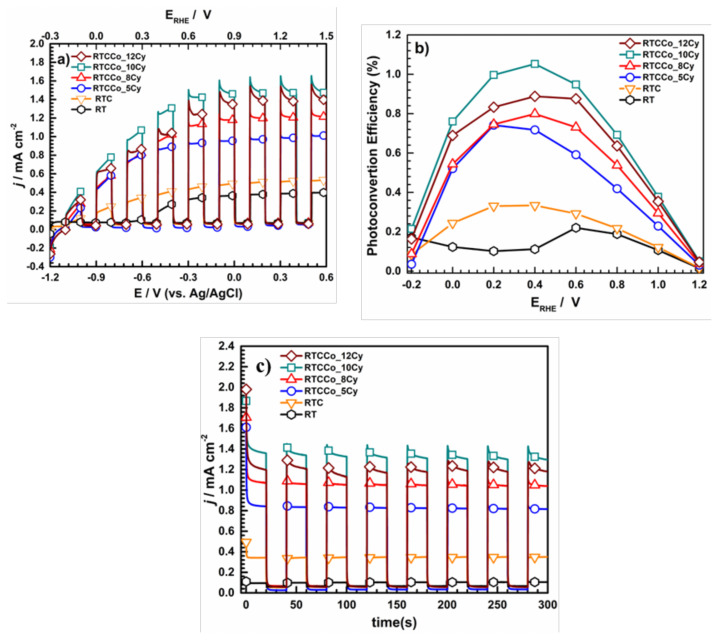
LSV results (a) photoconversion efficiency (b) and chronoamperometric measurement of photoelectrodes.

LSV, ABPE and *j-t* were conducted to enlighten on the sample’s photocatalytic activity for solar-driven water splitting to produce hydrogen. The LSV measurements were performed −1.2–0.6 V (vs. Ag/AgCl) under the chopped method, which is given in Figure 5 (a). The chopped procedure clearly indicates zero current density and rapid current density increasing, dark and light conditions, respectively. It could be concluded that all the obtained samples act as a semiconductor under photo-induced by solar light. The electrochemical water electrolysis overall potential is about 1.23 V (vs. RHE), which is critical to distinguish the photochemical performance of electrodes. Therefore, As comparing current density at the 1.23 V (vs. RHE), RT, RTC, RTC_5Cy, RTC_8Cy, RTC_10Cy and RTC_12Cy, are 0.377 mA cm^−^^2^, 0.522 mA cm^−^^2^, 1.013 mA cm^−^^2^, 1.284 mA cm^−^^2^, 1.534 mA cm^−^^2^ and 1.647 mA cm^−^^2^, respectively. It indicates that i) CdS QDs enhanced photo response of RT as a result of further photo-excited electron of CdS giving to RT energy level, ii) Co-doped CdS QDs enhances photocatalytic activity compared to without Co-doped CdS QDS, due to Co^2+^ doped enable further rapid charge recombination on CdS QDs, iii) increasing SILAR cycle number improve catalytic activity on water splitting, but the current density of RTC_12Cy decreases relating excess Co-doped CdS QDs loading blocked of active surface on RT surface.

In the photoelectrochemical setup, hydrogen production was obtained on the photoelectrode surface by photoelectrochemical reaction, which is associated with both bias potential and solar irradiation applied to the faradaic process. Therefore, one of the most photoconversion efficiency of the electrode is ABPE, given in Figure 5 (b). The measured maximum photoconversion efficiency % of RT, RTC, RTCCo_5Cy, RTCCo_8Cy, RTCCo_10Cy and RTCCo_12Cy, are 0.221, 0.334, 0.742, 0.799, 1.053, and 0.888, respectively. The results indicate that RT photoconversion efficiency % is not only enhanced with CdS QDS but also with various cycles Co-doped CdS QDs electrodes are further improved photocatalytic activity on water splitting. It relates to Co-doped enabling electrical conductivity, increasing RT electrode and leading to more electron transfer among CdS QDs energy level [56]. Consequently, RTCCo_10Cy demonstrates the utmost photoconversion efficiency %, and Co-doped CdS QDs at 12 cycles lead to decreasing photoconversion efficiency % value. It suggests increasing the number of cycles causing the block to active side on the RT surface and decreasing imposing solvated ion at the electrode/electrolyte boundary by the photoelectrochemical process.

The chronoamperometric measurement was performed to photo-durability under a bias 0.3 V (vs. RHE) potential with the chopped method, as shown in Figure 5 (c). The light-off condition demonstrates zero current density due to inaccessible electron transition among semiconductor energy levels. The samples perform abrupt current density increasing and achieve a steady state under light-on conditions, resulting in rapid electron decay from VB to CB band and electron/hole recombination procedure, respectively. As seen in Figure 5 (c), all samples are performed durable photoresponse under the chopped method after about two s for 300 s. Furthermore, the chronoamperometric curve implies that Co-doped among various cycles CdS QDs electrodes RTCCo_10Cy shows the highest current density, which conforms with the LSV results.

**Figure 6:**
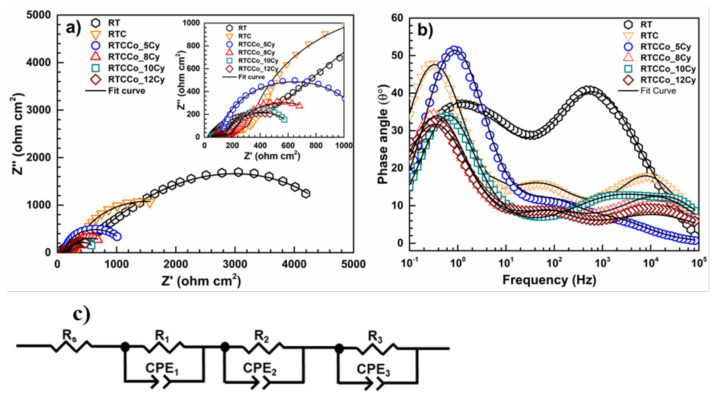
Nyquist plot (a), phase angle-frequency plot (b), and suggested electrical equivalent circuit (c).

Commonly, EIS measurement prefers to recognize electrode/electrolyte interface resistances, electron/hole recombination process, and photoelectrochemical charge transfer [57–59]. To understand resistance sources connected with the photoelectrochemical reaction, EIS measurements were carried out with an applied bias potential of 0.3 V (vs. RHE) under 100 mW cm^−^^2^. Nyquist and phase angle-frequency plots of the photoelectrodes are shown in Figure 6(a and b). According to the Nyquist plot, electrodes display three depressed loops, two of which are in the higher frequency region and one of which is in the lower frequency region. Based on the EIS theory, a smaller loop denotes a lower resistance, whereas a larger loop leads to a higher resistance at the electrode-electrolyte double layer. Both CdS QDs and Co doped CdS QDs deposition leads decreasing in depressed loops RT, suggesting photocatalyst loading on RT reduced various resistances sources on O_2_ evolution reaction (OER). Additionally, when compared to other samples, RTCCo_10Cy represents the lowest loop, which enhances the electrical conductivity of the Co-doped CdS QDs and provides more electrons from the VB to CB energy level. Phase angle-frequency plots of the samples are given in Figure 6 (b). According to phase angle-frequency plots, all samples indicate three-time constants from higher to lower frequency. The last point of the curve on the phase angle-frequency plot indicates the R_f_ and indicator coverage of the electrode at the lower frequency. The applying Co-doped process assists in decreasing of R_f_ value, resulting in electron transportation among energy levels of Co, providing reduce the speed of the recombination process and the electrons remain for a slower time in the Co orbitals at the lower frequency region. Furthermore, Co-doped provides decreasing in maximum phase angle on RT and RTC electrodes.

EIS measurement was fitted to calculate electrochemical parameters by a suggested electrical equivalent circuit (Figure 6). The electrochemical parameters are listed in Table 1, including various resistances and constant phase elements (CPE). The electrical equivalent circuit diagram demonstrates that the samples consist of three CPE. R_1_ is the charge transfer resistance (R_ct_) (at higher frequency region), R_2_ is the barrier resistance (R_b_), and R_3_ is the film resistance (R_f_) (at lower frequency). It refers to R_s_, which represents to the solution resistance (related uncompensated resistance). The sum of all resistance in the photoelectrochemical cell is called the polarization resistance (R_p_), including R_ct_, R_b,_ and R_f_. According to Table 1, when compared to samples of undoped CdS and unmodified RT, the R_p_ values of Co-doped CdS QDs modified RT are the lowest. It suggests that Co is doped on the surface, enabling decreasing resistance on OER process sources at the electrode/electrolyte interface. Furthermore, RTCCo_10Cy demonstrates the lowest R_p_ among RTCCo electrodes, suggesting that the optimum Co-doped in the CdS QDs is 10 SILAR cycles, leading to imposed active surface boundary to photocatalytic reaction. Increasing of SILAR cycles to 12 caused to blocked catalytic surface and rising R_f_ value on the OER process.

**Figure 7:**
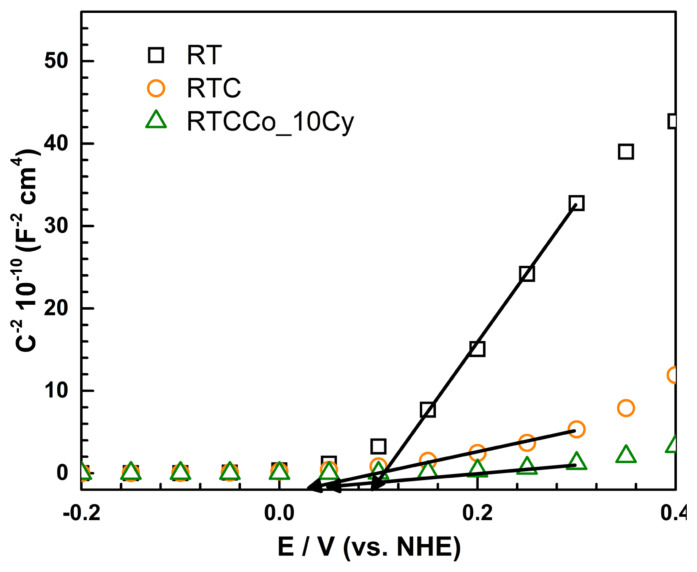
The Mott-Schottky plots of photoelectrodes.

A semiconductor/electrode interface can be measured by Mott-Schottky measurement. Figure 7 demonstrates the Mott-Schottky plot of samples ranging from −0.2 and 0.4 V. A negative slope and positive slope indicate a p-type and an n-type semiconductor, respectively. As seen in Figure 7, all samples show an n-type semiconductor. The theory of capacitance provides a calculation of charge carrier density (*N_D_*) at the electrochemical double layer by using the following Mott-Schottky equation [60];


(4) $$\frac{1}{C^{2}}=\frac{2}{qɛ\;{ɛ}_{0}\;N_{D}}\left[V-V_{fb}-\frac{K_{B}\;T}{q}\right]$$

Herein *q* is the electron charge, *C* is the capacitance at the electrochemical double layer, *ε*_0_ is the permittivity in a vacuum, ε is the dielectric constant, *k_B_* is the Boltzmann constant, *V* is the applied bias potential, *V_fb_* is the flat band potential and *T* is the condition temperature. The *V_fb_* value of the sample calculates with an extrapolation curve to the x-axis. Furthermore, the electron density *N_D_* obtains on curve slope (S) by following equation [61];


(5) $$N_{D}=\frac{2}{qɛ\;{ɛ}_{0}\;S}$$

The *N_D_* and *V_fb_* of samples are given in Table 2. The *N_D_* is associated with the steady state of the semiconductor, performing no charge depletion or band bending. According to obtained *N_D_*, RTCCo_10Cy performs the utmost value, suggesting provides further electron transportation to photoelectrochemical water splitting at the double layer. Increasing C corresponds to the charging of electrode/electrode boundary to nonfaradaic processes. Therefore, the *C* value is the lowest at 0.4 V compared to RT and RTC. The *V_fb_* corresponds to the low velocity of electron/hole recombination at the energy levels of the semiconductor. By way of the *V_fb_* further negative, electron/hole recombination rate decreases and performs catalytic photoresponse. As seen in Table 2, RTCCo_10Cy shows more negative *V_fb_* value, concluding high photocatalytic performance on the OER process.

## 4. Discussion

By using the SILAR cycle deposition process, we have successfully produced the nanoparticles of CdS QDs and Co-doped CdS QDS on RT. The cycle effect of SILAR deposition was analysed in detail on the crystal structure, surface morphology, light absorption, and photoelectrochemical performance. The XRD patterns confirmed distinctive 20 ° peaks of CdS are 26.72 ° and 44.33 °. FESEM surface images showed that increasing SILAR cycles caused to enhancing deposition of Co-doped CdS QDS on RT and coverage to the active side of the boundary. EIS measurement revealed RTCCo_10Cy’s lowest loop compared to other samples, resulting in enhancing the electrical conductivity. Mott-Schottky measurement showed that all samples were an n-type semiconductor behaviour and the RTCCo_10Cy electrode exhibited the major *N_D_* and the most negative *V_fb_* The photoelectrochemical measurements revealed CdS QDs and Co doped CdS QDS enhanced photoelectrochemical response of RT and RTCCo_10Cy the highest under solar irradiation. The present study demonstrates as an optimum Co doped CdS QDS SILAR deposition cycle is 10 cycles on RT photoelectrode revealing superior photocatalytic properties which can be applied to solar cells and other photocatalytic applications.

## Figures and Tables

**Table 1:** Electrochemical parameters of photoelectrode by fitted Zview software.

Photoelectrode	R_1_ Ω cm^2^	C_CPE1_ × 10^−4^ Ω^−1^s^n^cm^−2^	R_2_ Ω cm^2^	C_CPE2_ × 10^−4^ Ω^−1^s^n^cm^−2^	R_3_ Ω cm^2^	C_CPE3_ × 10^−4^ Ω^−1^s^n^cm^−2^	R_p_ Ω cm^2^
RT	435	0.302	1698	3.992	2960	1.481	5093
RTC	78	0.578	194	1.678	2401	5.997	2673
RTCCo-5Cyc	45	2.334	13	46.141	1050	6.101	1108
RTCCo-8Cyc	71	3.080	66	51.980	725	11.417	862
RTCCo-10Cyc	98	4.173	8	53.181	511	12.011	617
RTCCo_12Cyc	33	1.582	46	48.454	580	21.414	638

**Table 2:** Mott-Schottky parameters of photoelectrodes.

Electrodes	*V_fb_* (V_RHE_)	N_d_ × 10^18^ cm^−3^
**RT**	0.168	0.492
**RTC**	0.098	2.543
**RTCCo_10 Cyc**	0.120	14.603
